# Lithium Inhibits Tumorigenic Potential of PDA Cells through Targeting Hedgehog-GLI Signaling Pathway

**DOI:** 10.1371/journal.pone.0061457

**Published:** 2013-04-23

**Authors:** Zhonglu Peng, Zhengyu Ji, Fang Mei, Meiling Lu, Yu Ou, Xiaodong Cheng

**Affiliations:** 1 State Key Laboratory of Natural Medicines and School of Life Science and Technology, China Pharmaceutical University, Nanjing, China; 2 Department of Pharmacology and Toxicology, The University of Texas Medical Branch, Galveston, Texas, United States of America; Indiana University School of Medicine, United States of America

## Abstract

Hedgehog signaling pathway plays a critical role in the initiation and development of pancreatic ductal adenocarcinoma (PDA) and represents an attractive target for PDA treatment. Lithium, a clinical mood stabilizer for mental disorders, potently inhibits the activity of glycogen synthase kinase 3β (GSK3β) that promotes the ubiquitin-dependent proteasome degradation of GLI1, an important downstream component of hedgehog signaling. Herein, we report that lithium inhibits cell proliferation, blocks G1/S cell-cycle progression, induces cell apoptosis and suppresses tumorigenic potential of PDA cells through down-regulation of the expression and activity of GLI1. Moreover, lithium synergistically enhances the anti-cancer effect of gemcitabine. These findings further our knowledge of mechanisms of action for lithium and provide a potentially new therapeutic strategy for PDA through targeting GLI1.

## Introduction

Pancreatic ductal adenocarcinoma (PDA), characterized by extreme aggressiveness, poor prognosis and high lethality, stands as the fourth leading cause of cancer-related death in the United States and shows little improvement in survival over the past 30 years [1]. PDA is reflective to current chemotherapeutic treatments as agents effective for other cancer types offer very limited survival benefit for PDA patients [2], [3], [4], [5], [6]. Surgical resection and gemcitabine chemotherapy are the main clinical treatment options for PDA patients based on the stage of diagnosis. Five-year relative survival rate for ∼20% of the PDA patients feasible for surgical resection is less than 20%, while the five-year relative survival rate of all stages patients is less than 6% [1], [7]. Therefore, a better understanding of PDA pathophysiology and the development of novel therapeutic options are urgently needed.

Hedgehog signaling pathway (Hh pathway), initially discovered in *Drosophila* to be important for the development of fruit fly body fragmentation, is a key regulator of animal development [8], [9]. This pathway in human starts with an intercellular ligand, hedgehog (HH) molecule, from autocrine and paracrine secretion. In the absence of HH ligand, a membrane receptor protein called patched (PTCH) represses the activity of another transmembrane receptor smoothened (SMO). Binding of HH ligand to PTCH releases the repression of SMO by the PTCH, and transduces the extracellular signal by activating downstream GLI zinc finger transcription factors 1 (GLI1), a hallmark of the activation of Hh pathway [10], [11].

Abnormal activation of the Hh pathway promotes the growth, proliferation, migration, invasion, angiogenesis and tumorigenic potential of cancer cells, and has been implicated in many human cancers [12], [13]. In pancreatic cancer patients, dysregulation of Hh pathway is not only present in PDA, but also in its precursor, pancreatic intraepithelial neoplasia (PanIN), suggesting that this pathway is an important early and late mediator of pancreatic cancer tumorigenesis [14]. Moreover, abnormal activation of the Hh pathway can be enhanced and sustained by mutations in key components of the canonical Hh pathway or by abnormal HH ligand in tumor microenvironment, as well as from noncanonical “cross talking” between Hh pathway and other pathways, such as the RAS/RAF/MEK/ERK pathway [15], [16]. Aberrant Hh pathway plays critical roles in the occurrence and development of epithelial mesenchymal transition (EMT) [17], oncogenic transformation and angiogenesis [18] in PDA. While suppression of Hh pathway by SMO inhibitors such as cyclopamine has been used as a therapeutic strategy for cancer, a significant fraction of GLI1 activation in PDA is driven by a SMO-independent mechanism [19], suggesting that direct inhibition of GLI1 protein may be a more effective route to suppress Hh pathway activation [20], [21] in PDA.

Lithium ions, a classical mood stabilizer, have been used in the clinical treatment of bipolar disorder and other mental disorders for more than half a century [22]. Lithium acts on a panel of molecular targets, majority of which are metal-dependent enzymes, such as glycogen synthase kinase 3 (GSK3α and GSK3β) [23], [24], [25], protein kinase B (PKB) [25], inositol monophosphatase (IMPase) [26], phosphoglucomutase [26] and bisphosphate 3′-nucleotidase (BPNT1) [27], presumably via direct competition with Mg^2+^ [28]. Although lithium is mainly used to treat mental disorders, it targets not only the nerve cells. Several reports have shown that lithium salts are effective for inhibiting glioma cell [29], colorectal cancer cell [30], medulloblastoma cell [31], hepatocellular carcinoma cell [32] and other cancer cells [33]. In fact, it has been suggested that lower cancer prevalence observed in mental patients is likely due to benefit from protection of lithium treatment [34]. At the molecular level, lithium has been shown to inhibit the growth and/or tumorigenicity of cancer cells by modulating the biologic activities of many cancer related genes, for example, STAT3 [35], β-catenin/WNT [30], [31], TNF [33] and FASL [36], and P53 [37].

In this study, we report that lithium inhibits cell proliferation and tumorigenic potential of PDA cells *in vitro* through suppressing the stability of GLI1 protein. Moreover, lithium synergizes the efficacy of gemcitabine on PDA. These novel findings expand our knowledge of lithium’s biological functions and provide a new therapeutic potential of lithium for the treatment of PDA.

## Results

### Lithium Inhibited Cell Proliferation and Impaired Tumorigenic Potential of PDA Cells

**Figure 1:**
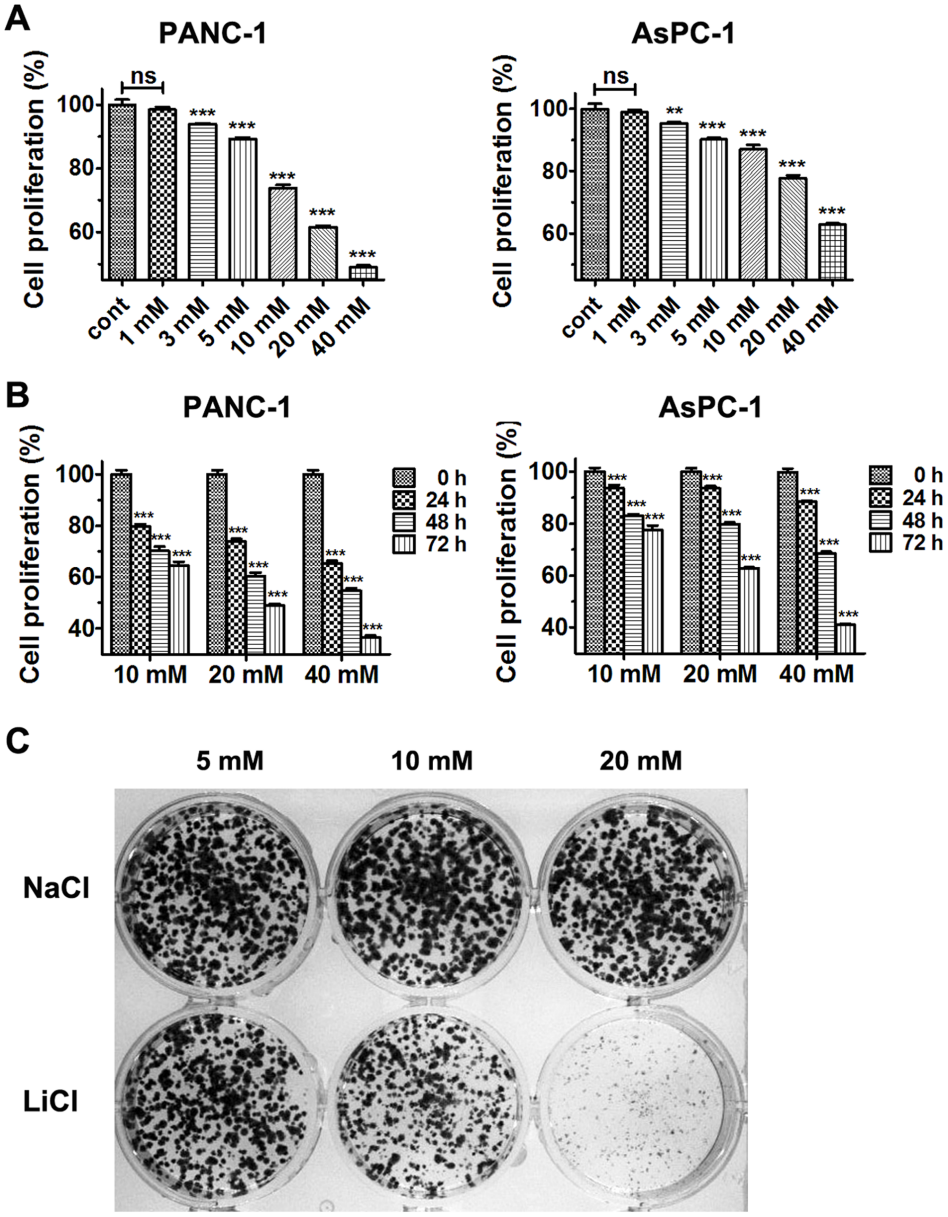
Lithium chloride suppresses the proliferation of PDA cells and impairs tumorigenic potential of PANC-1 cell. (A) Cell proliferation of PANC-1 cells and AsPC-1 cells after treatment with various concentrations of LiCl for 48 h. (B) Cell proliferation of PANC-1 cells and AsPC-1 cells after treatment with various concentrations of LiCl for indicated times. Data (Mean ± SEM) were representative of three independent experiments in triplicate. *P* values are indicated by asterisks (relative to control): *P<0.05, **P<0.01, ***P<0.001. (C) Tumorigenic potential of PANC-1 cells as monitored by colony formation after treatment with various concentrations of LiCl.

To investigate the effect of lithium on PDA cell proliferation, PANC-1 and AsPC-1 cells were treated with different doses of lithium chloride for one to three days and cells viabilities were determined. As shown in **Figure 1A & B**, treatment with lithium chloride, but not sodium chloride, markedly inhibited the proliferation of PANC-1 and AsPC-1 cells in a dose- and time-dependent manner. To further test if lithium treatment inhibits the tumorigenic potential of PDA cells, we performed colony formation assay, which is a test for oncogenic transformation *in vitro*. We observed that cell colonies were fewer and smaller for PANC-1 treated with lithium chloride than those from the control group with identical sodium chloride concentration in a doses-dependent manner (**Fig. 1C**).

### Lithium Induced Apoptosis and Cell Cycle Arrest of PDA Cells

**Figure 2:**
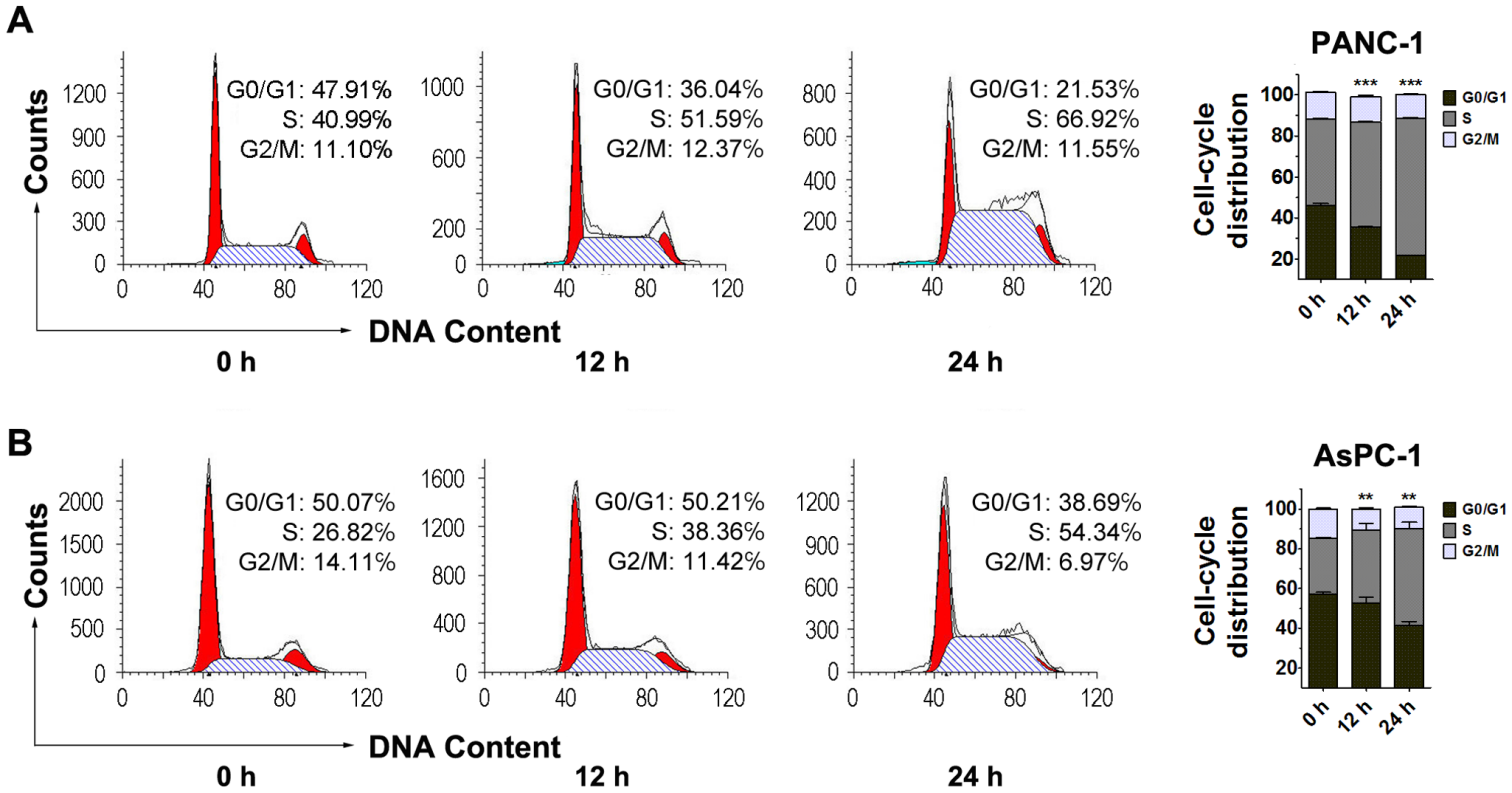
Lithium chloride induces cell cycle arrest in PANC-1 cells and AsPC-1 cells. PANC-1 cells (A) and AsPC-1 cells (B) were treated with 20 mM LiCl and analyzed by ﬂow cytometry with PI staining. Data (Mean ± SEM) were representative of three independent experiments. *P* values are indicated by asterisks (relative to control): *P<0.05, **P<0.01, ***P<0.001.

**Figure 3:**
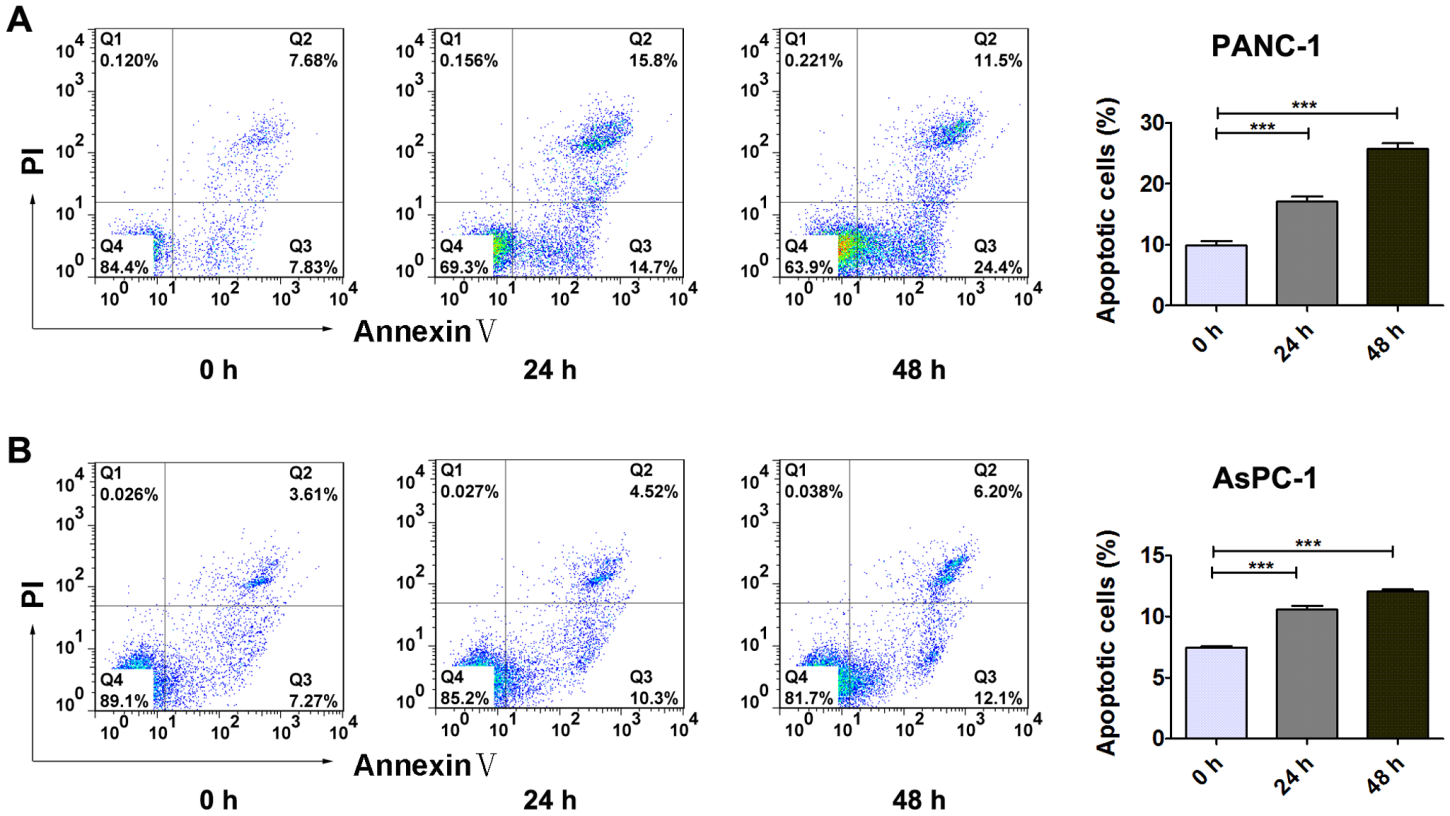
Lithium chloride induces apoptosis of PANC-1 cells and AsPC-1 cells. PANC-1 cells (A) and AsPC-1 cells (B) were treated with 20 mM LiCl and analyzed by flow cytometry with Annexin V-FITC/PI staining. Viable cells are Annexin V-FITC and PI negative (quadrant Q4), while apoptotic cells are Annexin V-FITC positive and PI negative (quadrant Q3); necrotic cells are Annexin V-FITC and PI positive (quadrant Q2), and damaged cells are Annexin V-FITC negative and PI positive (quadrant Q1). Data (Mean ± SEM) were representative of three independent experiments. *P* values are indicated by asterisks (relative to control): *P<0.05, **P<0.01, ***P<0.001.

To test the effects of lithium on cell cycle progression and survival of PDA cells, we performed flow cytometric analyses of PANC-1 and AsPC-1 cells after 12 hours and 24 hours treatment with 20 mM lithium chloride. As shown in **Figure 2**, lithium chloride treatment led to a significant increase in S-phase cell population and a decrease in population of G_0_/G_1_ phase in a time-dependent manner. Similarly, the percentage of apoptotic PDA cells was increased by lithium chloride treatment in a time-dependent manner (**Fig. 3**). These data suggested that growth inhibition of PDA cells by lithium was at least partly due to S-phase cell cycle arrest and apoptosis.

### Lithium Repressed Hedgehog Signaling Activity

**Figure 4:**
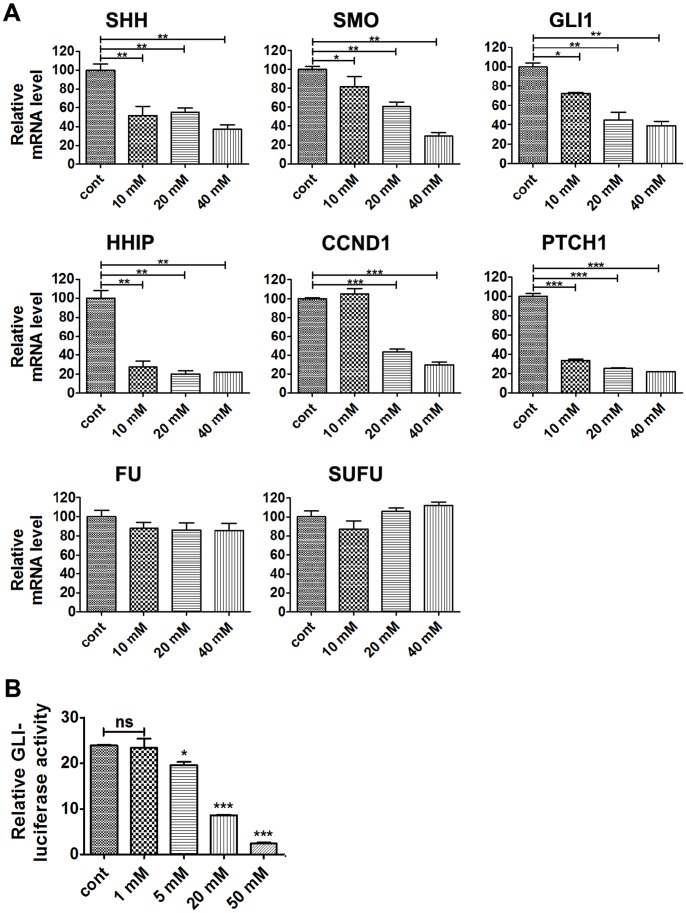
Lithium chloride represses Hh signaling activity. (A) Real-time PCR analysis of mRNA expression levels of components of the Hh signaling pathway in PANC-1 cells treated with different concentrations of LiCl for 24 h. Data (Mean ± SEM) were representative of three independent experiments. *P* values are indicated by asterisks (relative to control): *P<0.05, **P<0.01, ***P<0.001. (B) GLI-luciferase activities in AsPC-1 cells transfected with GLI-luciferase reporter and treated with different concentrations of LiCl for 18 h. Data (Mean ± SEM) were representative of three independent experiments. *P* values are indicated by asterisks (relative to control): *P<0.05, **P<0.01, ***P<0.001.

Abnormal Hh pathway plays an important role in PDA initiation and development. To determine whether lithium suppresses PDA cell proliferation through modulating the activity of Hh pathway, PANC-1 cells were treated with different concentrations of lithium chloride (10 mM, 20 mM, 40 mM) for 24 hours, and real-time PCR was carried out to monitor the expression levels of *SHH*, *PTCH1*, *SMO*, *FU*, *SUFU*, *GLI1* and Hh pathway target gene *HHIP* and *CCND1*. As shown in **Figure 4A**, the mRNA levels of *HHIP*, *CCND1* and *PTCH1* were markedly decreased, suggesting that the activity of Hh pathway was downregulated. This is consistent with the fact that the expression of *SHH*, *SMO* and *GLI1*, the positive regulators of Hh pathway, were also significantly reduced. On the other hand, the expression of *FU* and *SUFU* were not significantly influenced by lithium treatment. To determine if lithium was capable of repressing Hh pathway directly, GLI-mediated luciferase activity was monitored, and the results showed that GLI-luciferase activities decreased in response to lithium treatment in a doses-dependent manner in AsPC-1 cells (**Fig**. **4B**).

### Lithium Modulated the Cellular Protein Levels of GLI1

**Figure 5:**
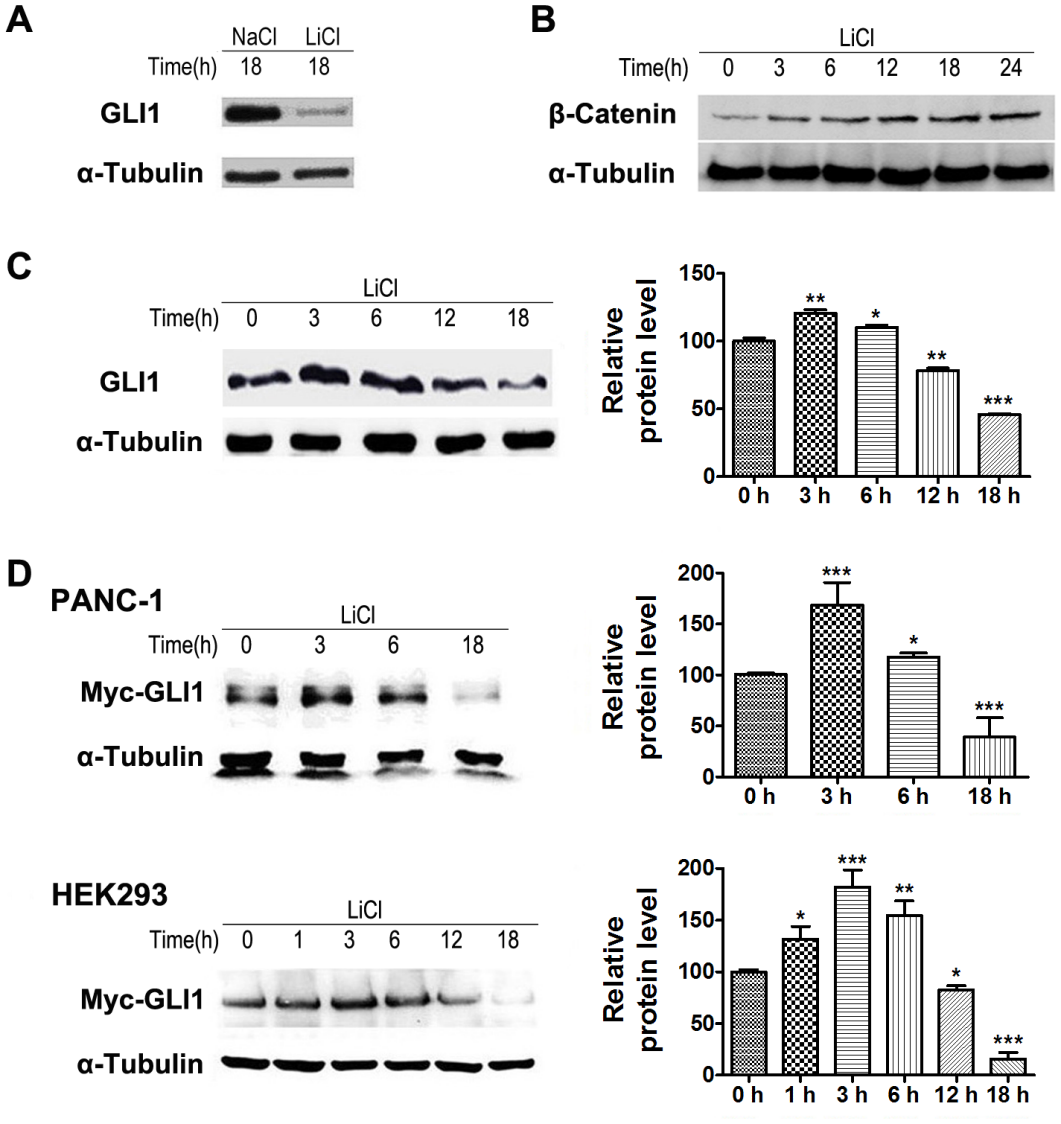
Lithium chloride regulates cellular levels of GLI1. (A) Levels of GLI1 protein in PANC-1 cell treated with 20 mM LiCl or 20 mM NaCl for 18 h. (B) Time-dependent accumulation of β-Catenin protein level in PANC-1 cell treated with 20 mM LiCl. (C) Time-dependent change of GLI1 protein level in PANC-1 cell treated with 20 mM LiCl. (D) Time-dependent change of Myc-GLI1 expression in PANC-1 cells and HEK293 cells treated with 20 mM LiCl. These cells were treated with LiCl for indicated time. Data (Mean ± SEM) were representative of three independent experiments. *P* values are indicated by asterisks (relative to control): *P<0.05, **P<0.01, ***P<0.001.

To determine if lithium suppressed Hh pathway through modulating the cellular expression of GLI1, endogenous GLI1 proteins in PANC-1 cell were monitored using anti-GLI1 antibody. Immunoblotting analysis showed that endogenous GLI1 level was decreased in PANC-1 cell after 18 hours of treatment with 20 mM lithium chloride (**Figure 5A**). Since lithium is an inhibitor of glycogen synthase kinase 3 beta (GSK3β) that is known to suppressed Hh pathway by promoting ubiquitin/proteasome-dependent degradation of GLI1, our results were quite unexpected as logically lithium should up-regulate GLI1 expression by inhibiting GSK3β. Hence, we followed the time-dependent dynamic expression of GLI1 and β-catenin in response to lithium treatment. While the level of β-catenin was increased persistently as expected (**Fig. 5B**), the endogenous GLI1 protein levels showed a biphasic expression pattern. They were up-regulated initially at 3 and 6 hours but down-regulated subsequently in PANC-1 cell (**Fig. 5C**). To further confirm the dynamic effects of lithium on modulating cellular levels of GLI1, we ectopically expressed the full-length GLI1 tagged with a C-terminal Myc epitope in PANC-1 and HEK293 cells. These cells were incubated with 20 mM lithium chloride 48 hours post-transfection, and the expression level of GLI1-Myc was monitored using anti-Myc-Tag antibody. As shown in **Figure 5D**, the expression of GLI1-Myc protein in both of PANC-1 and HEK293 cells followed a similar biphasic pattern as the endogenous GLI1. Taken together, our results suggest that lithium treatment promotes the degradation of GLI1 protein, consequently leads to the inactivation of Hh pathway in PDA cells.

### Lithium Synergized with Gemcitabine’s Suppressive Effects on Cell Viability and Tumorigenic Potential of PDA Cells

**Figure 6:**
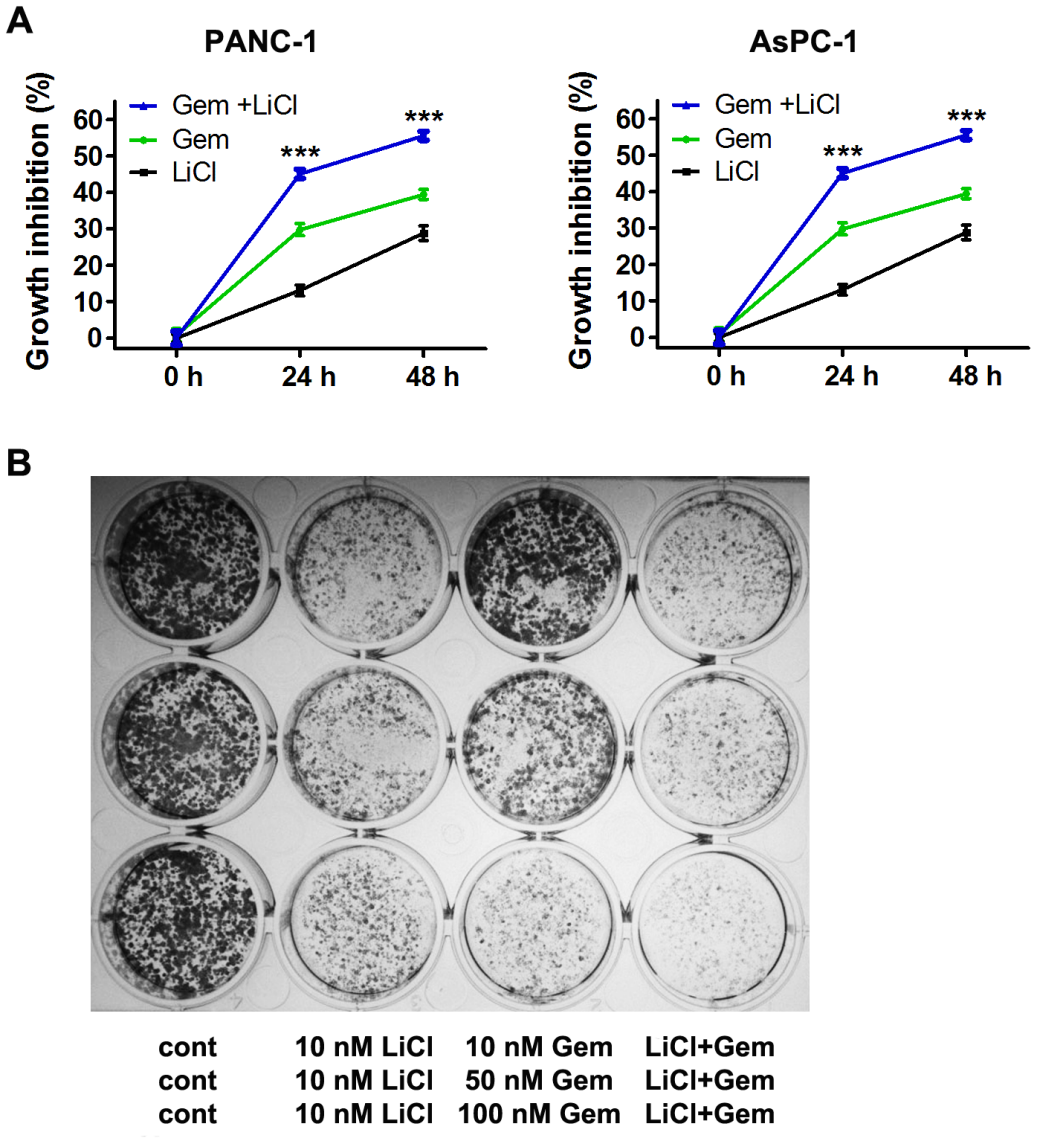
Lithium chloride synergizes with gemcitabine in reducing cell viability and tumorigenic potential of PDA cells. (A) Growth inhibition of PANC-1 and AsPC-1 cells after treatment with 20 mM LiCl, 200 nM gemcitabine and combination of LiCl and gemcitabine. Data (Mean ± SEM) were representative of three independent experiments. *P* values are indicated by asterisks (relative to control): *P<0.05, **P<0.01, ***P<0.001. (B) Tumorigenic potential of PANC-1 cells as monitored by colony formation after treatment with LiCl, gemcitabine and combination of LiCl and gemcitabine.

To determine if lithium-mediated Hh pathway suppression can synergize with gemcitabine to inhibit PDA cell proliferation, we followed cell viabilities of PANC-1 and AsPC-1 cells after treatment with lithium chloride (20 mM), gemcitabine (200 nM) or lithium chloride plus gemcitabine. The cell viabilities of both cell lines were significantly reduced by the combination treatment as compared to those of single agent treatment (**Fig. 6A**). Furthermore, the effect of lithium and gemcitabine combination on the tumorigenic potential of PANC-1 cell was investigated by colony formation assay. While gemcitabine dose-dependently suppressed the number and size of PANC-1 colonies, lithium significantly enhanced inhibitory effect of gemcitabine (**Fig. 6B**). These data suggest that lithium synergizes with gemcitabine’s suppressive effects on cell viability and tumorigenic potential of PDA cells.

## Discussion

The development of PDA is associated with the accumulation of genetic mutations and abnormal signaling pathways, including the KRAS, JAK/STAT, EGF, TGF-β/SMAD and Hh pathway [38], [39], [40]. Hh pathway inhibition has been proven to be an effective anti-cancer therapeutic strategy, and several antagonists targeting SMO have been developed and show efficacy in preclinical studies and clinical trials in humans [41], [42], [43], [44]. Recent studies demonstrate that it is sufficient to inhibit the growth of PDA through blocking GLI1 activity with RNAi technology or medicinal compounds [19], [45]. GLI1, can be phosphorylated by cAMP-dependent protein kinase (PKA), casein kinase I (CKI) and GSK3β, which in turn results in β-TRCP-mediated protein degradation by the ubiquitin-proteasome system [46], [47], which provides us a direction by targeting GLI1 on PDA therapy. GSK3β is a proline-directed serine-threonine kinase, involved in many cellular processes, such as metabolism, neuronal development, and body pattern formation [48], and GSK3β signaling has also been implicated in mental illness and tumor formation. Lithium, an effective GSK3β inhibitor, has been used in treating depression and bipolar disorder for many years [22]. Recently, it has been shown that inhibition of GSK3β promotes apoptosis in glioma cells and PDA cells [49], [50], and sensitizes PANC-1 cells to gemcitabine [51]. In addition, lithium induces apoptosis of a variety of cancer cells [36], [52]. At present, the mechanism of lithium-mediated anti-cancer activity is not clear.

In this study, we investigated the effect of lithium on Hh pathway in PDA cells. To our surprise, our results showed that the expression and activity of GLI1 in PDA cells were significantly down-regulated after treatment with lithium for 18 hours. Since GSK3β is known to promote ubiquitin-proteasome mediated GLI degradation, one would expect that inhibition of GSK3β by lithium should up-regulate cellular GLI levels. A more careful analysis revealed a biphasic regulation in which GLI1 protein levels were indeed increased initially following lithium treatment up to 6 hours. On the basis of these observations, coupled with the fact that in addition to directly phosphorylate GLI1, GSK3β is also known to control protein translation through direct suppression of EIF2β [53] and indirect suppression of MTOR [54], we deduce that the inhibition of GSK3β by lithium increases GLI1 cellular levels initially via blocking ubiquitin-proteasome mediated GLI degradation and releasing the inhibition of protein synthesis. At present, the long-term inhibitory effect of lithium on GLI1 is not clear. While we could not completely rule out the possibility that lithium-induced reduction of GLI1 over longer time course may be an indirect consequence of SHH and SMO downregulation (**Fig. 4A**). However, this scenario is most unlikely based on existing literature. Nolan-Stevaux and colleagues report that multistage development of PDA tumors is not affected by the deletion of *Smo* in the pancreas, suggesting a Smo-independent mechanism in which autocrine Shh–Ptch–Smo signaling is not required in pancreatic ductal cells for PDA progression [19]. This finding, coupled with the fact that more than 50% of PDA cells lines with sustained Hh signaling activity are resistant to SMO antagonist cyclopamine [14], implicates alternative means of GLI regulation by KRAS and TGF-β in PDA [15], [19]. Nevertheless, parallel observation of dual regulation involving GSK3β inhibition has been reported. Sun *et al.* show that 24-hour lithium treatment blocks prostate cancer cells at the S phase while lithium treatment for 6 hours promotes cells to pass through S phase [55]. Since GSK3β also promotes the degradation of E2F target gene cyclin E (CCNE) [56], it is most likely that CCNE may be also transiently increased at the beginning of lithium treatment, which facilitates the G1/S transition. On the other hand, it has been shown that long-term GSK3β inhibition by GSK3β specific inhibitor TDZD-8, lithium, or *GSK3β* siRNA disrupts the E2F-DNA interaction and suppresses the expression of E2F target genes *CDC6*, cyclin A (*CCNA*), *CCNE* and *CDC25C* [55]. Down-regulation of these DNA-replication related gene leads to G1/S arrest as reported and also shown in our study. Since *E2F* is a known target gene of GLI [57], the reported effects of lithium on E2F suppression and the cell cycle may depend on down-regulation of GLI1. We are currently actively testing this hypothesis.

In summary, we show for the first time that lithium inhibits Hh pathway through down-regulation of cellular GLI1 such that it blocks cell proliferation, induces cell-cycle arrest, promotes apoptosis and reduces tumorigenic potential of PDA cells. Moreover, lithium synergistically enhances the anti-cancer effect of gemcitabine. These novel findings extend our knowledge of mechanisms of action for lithium and provide a potentially new therapeutic strategy for PDA.

## Materials and Methods

### Cell Culture and Transfection

PDA cell lines PANC-1 and AsPC-1, and HEK293 were obtained from the Cell Bank of Type Culture Collection of Chinese Academy of Science (Shanghai, China). PANC-1 and HEK293 cell lines were cultured in DMEM and AsPC-1 cell line was in RPMI-1640 medium (Gibco/BRL) supplemented with 10% fetal calf serum (PAA, Austria) at 37°C under 5% humidified CO_2_. Transfection was performed using Lipofectamine 2000 reagent (Invitrogen) according to the manufacturer’s instructions.

### Cell Viability Analysis

Growth-phase PDA cells were seeded in 96-well plates at a density of 4×10^3^ cells/well (PANC-1) or 1×10^4^ cells/well (AsPC-1), respectively. The cells were treated with various concentration of LiCl (Calbiochem) and gemcitabine (Tocris) the next day for 0–3 days and six replications were performed for each treatment. Ten microliters of Cell Counting Kit-8 (CCK-8) solution (Dojindo Laboratories) were added to each well and incubated at 37°C for 2–6 hours. The absorbance of each well at 450 nm was determined using a plate reader and the growth curves were then plotted accordingly. Each experiment was performed independently three times.

### Colony Formation Assay

Single cell suspension of growth-phase PANC-1 cell was seeded in 6-well plates (1×10^3^ cells/well) or 12-well plates (5×10^2^ cells/well). After 24 hours incubation, cells were treated with various concentrations of LiCl and gemcitabine. Fresh medium with corresponding concentration of LiCl and gemcitabine were added every 5 days to replace the old medium. Two weeks later, the medium was aspirated off and the cells colonies were washed with PBS twice, then fixed for 15 minutes with methanol and stained with crystal violet (0.1% crystal violet in 20% methanol), and visualized using a microscope.

### Apoptosis and Cell Cycle Analyses

PANC-1 and AsPC-1 cells were distributed into 12-well plates at a density of 2×10^5^ cells/well, incubated with 20 mM LiCl. At the indicated time, cells were trypsinized and harvested by centrifugation, washed once with a buffer containing 10 mM Hepes, 140 mM NaCl, 2.5 mM CaCl_2_ and stained with Annexin V-FITC and propidium iodide (PI) in the dark at room temperature for 15 minutes, then analyzed using by flow cytometry (FACScan; Becton-Dickinson). Data were analyzed using the FlowJo software. For cell cycle analysis, the DNA contents of cells stained with PI and determined by ﬂow cytometry. Data were analyzed using the ModFit software package. Each experiment was performed independently at least three times.

### Immunoblotting Analysis

Cells were seeded in 6-well plates (5×10^5^ cells/well) and treated with LiCl the next day. At the indicated time, lysis buffer containing 62.5 mM Tris pH 6.8, 2% sodium dodecylsulfate, 10% Glycerol, 0.01% bromophenol blue (300 µl/well) was used to harvest cellular proteins. The protein samples from lysates with 2% β-mercaptoethanol added were separated using 8% sodium dodecylsulfate–polyacrylamide gel electrophoresis (SDS-PAGE) and transferred onto polyvinylidene difluoride (PVDF) membranes (Millipore). After being blocked with 5% non-fat dry milk in TBST (Tris buffered saline buffer containing 0.1% Tween-20) at room temperature for 1 hour, the PVDF membranes were incubated with individual primary antibodies (anti-Myc-Tag, anti-GLI1, Cell Signaling Technology; anti-α-Tubulin, Sigma; anti-β-catenin, Santa Cruz) at 4°C overnight, followed by corresponding HRP-conjugated secondary antibody at room temperature for 45 minutes. Protein bands were detected by ECL Western Blotting Detection System (Millipore). Each experiment was performed independently at least three times.

### Real-time PCR Assay

Total RNA was extracted from PANC-1 cell treated with LiCl at different concentrations using TRIzol reagent (Invitrogen). cDNA was synthesized using the TaKaRa RNAiso Reagent (TaKaRa) according to the manufacturer’s instructions. Real-time PCR for *GAPDH*, *GLI1*, *SHH*, *HHIP*, *PTCH1*, *SMO*, *FU*, *SUFU* and *CCND1* was performed on an Applied Biosystems stepone plus Sequence Detection System (Applied Biosystems) using SYBR green dye and primers as described in Table 1. For quantification, the relative mRNA level of specific gene expression was calculated using the 2^−ΔΔCt^ method with *GAPDH* level as the control. Each experiment was performed independently at least three times.

**Table 1:** Primers for Real-time PCR.

Genes	Forword primer (5′-3′)	Reverse primer (5′-3′)
*GLI1*	CTCCCGAAGGACAGGTATGTAAC	CCCTACTCTTTAGGCACTAGAGTTG
*PTCH1*	TCGAGACCAACGTGGAGGAG	CCGAGTCCAGGTGTTGTAGG
*HHIP*	TTCCATACCAAGGAGCAACC	TCTTGCCACTGCTTTGTCAC
*SHH*	CGGGAAGAGGAGGCACCCCA	GTACTTGCTGCGGTCGCGGT
*CCND1*	AGAAGGAGGTCCTGCCGTCC	GGTCCAGGTAGTTCATGGCC
*FU*	ACTCTGAGCAGACTTTGCGGAG	CCATCCAAGACAACCTGCTGTG
*SUFU*	AGAGTGCCGCCGCCTTTACC	ACGGGCTGCATCTGTGGGTC
*SMO*	CAGGAGGAAGCGCACGGCAA	TGCAGCGCAGGAAGTCAGGC
*GAPDH*	TGTGGGCATCAATGGATTTGG	ACACCATGTATTCCGGGTCAAT

### Luciferase Reporter Gene Assay

GLI luciferase reporter construct (8×3′ GLI BSwt-luc) was kindly provided by Dr. Hisato Kondoh [58]. AsPC-1 cells were transiently transfected with the GLI-luc plasmid and the SV40-Renilla control plasmid using Lipofectamine 2000 (Invitrogen) according to the manufacturer’s instructions. Twenty-four hours post-transfection, cells were treated with various concentration of LiCl for 18 hours. Luciferase activity was determined with the dual luciferase reporter assay system (Promega Corporation, WI) according to the manufacturer’s instruction, and normalized to renilla luciferase activity as a control for transfection efficiency. Each experiment was repeated at least three times with similar results.

### Statistical Analysis

Results were presented as mean±S.E.M. Statistical significance between groups was analyzed by one-way ANOVA followed with the Student–Newman–Keuls multiple comparisons tests. A *P*-value of <0.05 was considered significant.
